# Increased Risk of Urinary Tract Cancer in ESRD Patients Associated with Usage of Chinese Herbal Products Suspected of Containing Aristolochic Acid

**DOI:** 10.1371/journal.pone.0105218

**Published:** 2014-08-29

**Authors:** Shuo-Meng Wang, Ming-Nan Lai, Alan Wei, Ya-Yin Chen, Yeong-Shiau Pu, Pau-Chung Chen, Jung-Der Wang

**Affiliations:** 1 Department of Urology, National Taiwan University Hospital, Taipei, Taiwan; 2 Department of Statistics, Feng Chia University, Taichung, Taiwan; 3 School of Medicine, Stony Brook University, Stony Brook, New York, United States of America; 4 Institute of Occupational Medicine and Industrial Hygiene, College of Public Health, National Taiwan University, Taipei, Taiwan; 5 Department of Public Health, National Cheng Kung University Medical College, Tainan City, Taiwan; 6 Departments of Internal Medicine and Occupational and Environmental Medicine, National Cheng Kung University Hospital, Tainan City, Taiwan; Centro Nacional de Investigaciones Oncológicas (CNIO), Spain

## Abstract

**Introduction:**

Both end-stage renal disease (ESRD) and urothelial cancer (UC) are associated with the consumption of Chinese herbal products containing aristolochic acid (AA) by the general population. The objective of this study was to determine the risk of UC associated with AA-related Chinese herbal products among ESRD patients.

**Methods:**

We conducted a cohort study using the National Health Insurance reimbursement database to enroll all ESRD patients in Taiwan from 1998–2002. Cox regression models were constructed and hazard ratios and confidence intervals were estimated after controlling for potential confounders, including age, sex, residence in region with endemic black foot disease, urinary tract infection, and use of non-steroidal anti-inflammatory drugs and acetaminophen.

**Results:**

A total of 38,995 ESRD patients were included in the final analysis, and 320 patients developed UC after ESRD. Having been prescribed Mu Tong that was adulterated with Guan Mu Tong (*Aristolochia manshuriensis*) before 2004, or an estimated consumption of more than 1–100 mg of aristolochic acid, were both associated with an increased risk of UC in the multivariable analyses. Analgesic consumption of more than 150 pills was also associated with an increased risk of UC, although there was little correlation between the two risk factors.

**Conclusion:**

Consumption of aristolochic acid-related Chinese herbal products was associated with an increased risk of developing UC in ESRD patients. Regular follow-up screening for UC in ESRD patients who have consumed Chinese herbal products is thus necessary.

## Introduction

Aristolochic acid nephropathy—a progressive form of renal interstitial fibrosis—was first reported in a group of young Belgian patients with end-stage renal disease in 1993, and was thought to be caused by the use of Chinese herbal medicines that contained aristolochic acid [1]–[3]. Aristolochic acid has been shown to be associated with urothelial cancer (UC) in many studies of clinical cases around the world, in animal models, and by the detection of aristolochic acid–DNA adducts in the kidney and ureteral tissues [4] [5], [6]. Prior studies observed increased risks of developing UC and ESRD in the general population in association with the consumption of Chinese herbal products [7] [8], and patients with ESRD have a higher incidence of malignancies than the general population [9]–[12]. We have noticed an extraordinarily high incidence of UC in uremic or ESRD patients in the past decade in Taiwan [13]–[16], but the reason for this remains unknown. Some researchers suggest that chronic bladder irritation, a decreased urinary washout effect, atrophic involution of the bladder, compound analgesic abuse [17]–[19], use of Chinese herbs [4], [5], [20], groundwater intake (arsenic exposure) [21], [22], and uremia per se [15], [16] may play roles in the development of UC. A report published by the International Agency in Research on Cancer (IARC) [23] stated that the risk factors associated with UC include analgesics (phenacetin), herbal usage (aristolochic acid), heavy metals (arsenic) and tobacco smoking.

Although the IARC classifies aristolochic acid as a group 1 carcinogen, to the best of our knowledge there have been no cohort studies that examine the association between urinary tract cancer and the use of herbs or herbal products containing aristolochic acid in ESRD patients. In March of 1995, Taiwan established the National Health Insurance (NHI) program, which covers more than 99% of the population [24]. The NHI routinely reimburses enrollees for the cost of prescribed medicines, including Chinese herbal products containing aristolochic acid, which were widely prescribed before being banned in December 2003. We thus used the NHI reimbursement database to conduct an ESRD population-based cohort study to examine the association between having been prescribed Chinese herbal products that contain substantial amounts of aristolochic acid, including Guan Mu Tong and Guang Fangchi, and the risk of urinary tract cancer, as well as the possibility of a dose–response relationship between the two.

## Materials and Methods

### Study Population

Established in Taiwan in March 1995, the National Health Insurance program (NHI) covers over 99% of the population residents [24]. Standard mixtures of Chinese herbal products (CHP) are included in the regular schedule of reimbursement. The National Health Research Institutes (NHRI) transformed the NHI reimbursement data into files suitable for use by researchers, and which contain detailed information about the usage of conventional drugs and CHP [25]. This study was conducted using ESRD patient data obtained from the database of approximately more than 22 million people enrolled in the NHI. The data collection period began in 1996, but became more comprehensive after January 1997. As noted above, the NHRI anonymized and converted the reimbursement data into research-ready files, called the National Health Insurance Research Database (NHIRD) [25]. The identification numbers of all the individuals in the database were doubly encrypted to ensure their privacy.

The dataset to which we had access provided detailed demographic data (including birth date and sex) and information regarding the health-care services provided for each patient, including all payments for outpatient visits, hospitalizations, and prescriptions, as well as where each patient lived. The data for each hospitalization contained up to five diagnoses that were coded according to the International Classification of Diseases, Ninth Revision (ICD-9) [26], all drugs prescribed and the doses (i.e., conventional medicines, including generic and commercial brands of acetaminophen and non-steroidal anti-inflammatory drugs, as well as Chinese herbal products), and the date of each prescription. During the study period (i.e., from January 1, 1998, to December 31, 2002), all prescribed medications were covered under the NHI of Taiwan, and no drug could be dispensed at a pharmacy without a doctor's prescription.

To select potential case subjects for this study, we first obtained the NHI catastrophic illness registry files for all patients who were diagnosed with end-stage renal disease from January 1, 1998, to December 31, 2002. Because all patients who are registered as having a catastrophic illness are exempt from all copayments, their data is very comprehensive and has been carefully validated. A diagnosis of urinary tract cancer or end-stage renal disease made by doctors and officials of the NHI is usually accurate: urinary tract cancer must be proven by tissue pathology, and is classified as cancer of the upper urinary tract, which includes the renal pelvis and ureter (ICD-9 codes 189.1 and 189.2, respectively) or bladder cancer (ICD-9 code 188). The database contains 38,675 Non-UTC and 839 UTC prevalent cases of end-stage renal disease that were diagnosed from January 1, 1998, to December 31, 2002.Within this population, we identified 320 patients who were newly diagnosed with urinary tract cancer from January 1, 2001, to December 31, 2002, to allow at least four years between January 1, 1997, and the date of diagnosis to give sufficient time for the case subjects to accumulate sufficient doses of herbal products to induce UTC.

### Exposure Assessment

The reimbursement database contained all the details of the prescribed conventional medicines, which included acetaminophen and the commercial names of 45 kinds of non-steroidal anti-inflammatory drugs (NSAIDs), shown in the File S1.

As phenacetin has been totally banned by the Department of Health since 1986, it was not included. Doses of each drug were determined according to the number of pills prescribed and cumulative doses were calculated before ESRD. The use of 600–1000 pills of acetaminophen, NSAIDs, or mixed analgesics has been associated with an increased risk of renal damage or renal cancer in previous studies [27], . We thus accumulated the total number of analgesics pills for each subject before dialysis during 1998–2002.

According to the standard prescription recommended by the Committee on Chinese Medicine and Pharmacy (CCMP) in Taiwan, the following Chinese herbal products may contain AA: Xi-Xin (Asarum heterotoppoides), Guan-Mu-Tong (Aristolochia manshuriensis), and Guang-Fangchi (A. fangchi). However, Guan-Mu-Tong and Guang-Fangchi were once offered under the names Mu-Tong (Akebia sp.) and Fangchi (Stephania sp.), respectively, in Taiwan before 2003, because of similarities of gross morphology and common practices [29]. In addition, according to an investigation by the Bureau of Food and Drug Analysis in Taiwan, as well as some studies, approximately 89.2–100% of Fangchi preparations were actually Guang-Fangchi [30]–[32] and 84% of Mu-Tong were actually Guan-Mu-Tong [33]. These three herbs were prescribed as single products or included as components of some mixed CHP. Each pharmaceutical company has published and submitted the detailed composition of every product it produces, and data on this can be retrieved from the website of the Committee on Chinese Medicine and Pharmacy of the Department of Health [29]. With this information, the original amounts of herbs, in grams, could be determined for each mixture of CHP, and the cumulative dose for each herb prescribed to an individual before developing ESRD could thus be calculated. We also calculated the estimated cumulative dose of aristolochic acid for each subject by using the following estimates obtained in previous studies: the estimated average doses of aristolochic acid per 1 g of Guan Mu Tong, Guang Fangchi, and Xi Xin are 2.59 mg, 2.04 mg, and 0.042 mg, respectively [30], [32]–[35].

The reimbursement database also has data on where all the subjects lived. We identified subjects who lived in the four townships in Taiwan that have been reported to be areas endemic for black foot disease—Pu-Tai and Yi-Chu in Chiayi County, and Hsueh-Chia and Pei-Men in Tainan County [36], [37]. Black foot disease is a peripheral vascular disease that has been endemic to the coastal region of Taiwan for the past 60 years, and is associated with drinking water from artesian wells containing arsenic, and has been documented to be associated with an increased incidence of bladder cancer [36], [37]. We controlled for this factor (townships) as a surrogate for arsenic exposure.

According to the Committee on Chinese Medicine and Pharmacy [29], Mu Tong is usually prescribed for the treatment of hepatitis, urinary tract infection, rhinitis, dysmenorrhea, and eczema. Recurrent or chronic urinary tract infection, associated with Schistosomiasis or prolonged indwelling catheters in patients with spinal cord injury, is associated with an increase risk of bladder cancer [38], [39], whereas urinary tract infection from other causes does not show any consistent association with bladder cancer risk [38], [40]. We thus defined patients with chronic urinary tract infection (UTI) as those who had such a diagnosis at least 12 times up to one year before the diagnosis of UC, and we controlled for the above potential confounders during the risk-estimate analysis. The patients with diabetes or hypertension were also ascertained based on the related diagnosis numbers in ICD-9 before ESRD diagnosis.

### Statistical Analyses

To assess the independent association of various risk factors with new occurrences of UC, univariate and multivariable Cox regression models were used to analyze the population of ESRD patients and those cases that developed UC subsequent to ESRD diagnosis. Potential risk factors, including age, sex, hypertension, diabetes mellitus, chronic UTI, and prescriptions of NSAIDs, acetaminophen or any of the aforementioned Chinese herbs suspected to contain AA, were assessed for independent association with new occurrences of UC. We constructed models for two different types of exposure assessment: prescribed dosages of Chinese herbs (model 1) and different estimated dosages of AA as risk factors (model 2). The dose–response association between cumulative dose of Chinese herbs, analgesics and occurrence of UC was tested by the Mantel–Haenszel extension for the trend. For each potential risk factor, multivariate Cox proportional hazards models were constructed to estimate the relative risk and its 95% confidence interval (CI) for UC incidence. An estimate with the 95% CI that did not contain the number 1 was considered statistically significant. We also ran a correlation analysis between the total numbers of analgesics pills and cumulative doses (in mg) of AA. All the above analyses were conducted using the SAS ver. 9.2 software package (SAS Institute, Cary, NC, USA).

## Results

After excluding people with incomplete data or aged over 100 years, a total of 39,514 prevalent cases of end-stage renal disease were included in the data, with 839 of these developing UTC between January 1, 1998 and December 31, 2002. Among these patients, there were 38,675 cases without UTC and 320 UTC cases who were newly diagnosed with urinary tract cancer between January 1, 2001 and December 31, 2002. A total of 38,995 ESRD patients were thus included in the final analysis, with 18,522 (47.5%) men and 20,473 (52.5%) women. There were high prevalence rates of hypertension (88.5%) and diabetes mellitus (55.7%). The average crude incidence rate of UTC for these ESRD patients was 1,368 per million person-years.

Table 1 summarizes the frequency data of the ESRD patient population with respect to different potential risk factors, including sex, age, follow-up time after ESRD diagnosis, residence in township with endemic black foot disease, hypertension, diabetes, chronic UTI, and analgesics (NSAID and acetaminophen) consumption. The patient population was also characterized in terms of Chinese herb consumption for individual herbs (Mu-Tong, Fangchi, and Xi-Xin), as well as total estimated consumption of AA calculated based on total consumption of Chinese herbs containing this substance. The incidence rate of UTC appears to increase with the time after diagnosis of ESRD (Table 1).

**Table 1:** Frequency distributions of various risk factors for the occurrence of urinary tract cancers (UTC) stratified by different inclusion criteria in 38,995 patients with end-stage renal disease (ESRD).

	All ESRD Patients	
Risk Factors	UTC Cases (n = 320)	Non-UTC Cases (n = 38675)
Sex		
Men	131 (40.94%)	18391 (47.55%)
Women	189 (59.06%)	20284 (52.45%)
Age (year)		
<50	67 (20.94%)	9804 (25.35%)
50–59	80 (25.00%)	7790 (20.14%)
60–69	99 (30.94%)	10572 (27.34%)
70–99	74 (23.13%)	10509 (27.17%)
Residence in township where black foot disease was endemic		
No	319 (99.69%)	38421 (99.34%)
Yes	1 (0.31%)	254 (0.66%)
Hypertension		
No	68 (21.25%)	4297 (11.11%)
Yes	252 (78.75%)	34378 (88.89%)
Diabetes		
No	219 (68.44%)	17045 (44.07%)
Yes	101 (31.56%)	21630 (55.93%)
Chronic UTI		
No	312 (97.50%)	38511 (99.58%)
Yes	8 (2.50%)	164 (0.42%)
Analgesics *(pills) NSAID & acetaminophen		
0–150	123 (38.44%)	16116 (41.65%)
151–300	74 (23.13%)	8187 (21.16%)
351–450	32 (10.00%)	4405 (11.40%)
451–600	25 (7.81%)	2665 (6.90%)
>600	66 (20.63%)	7306 (18.88%)
Mu-Tong (g) total amount prescribed		
0	286 (89.38%)	35968 (93.00%)
1–30	20 (6.25%)	1845 (4.77%)
30–60	3 (0.94%)	348 (0.90%)
61–100	3 (0.94%)	179 (0.46%)
101–200	4 (1.25%)	164 (0.42%)
>200	4 (1.25%)	171 (0.44%)
Fangchi (g) total amount prescribed		
0	295 (92.19%)	35731 (92.39%)
1–30	19 (5.94%)	2417 (6.25%)
31–60 (31–100)	4 (1.25%)	271 (0.70%)
61–100	0 (0%)	121 (0.31%)
101–200 (>100)	2 (0.63%)	86 (0.22%)
>200	0 (0%)	49 (0.13%)
Xi-Xin (g) total amount prescribed		
0	283 (88.44%)	34679 (89.67%)
1–30	22 (6.88%)	2995 (7.74%)
31–60	5 (1.56%)	465 (1.20%)
61–100	5 (1.56%)	231 (0.60%)
101–200	3 (0.94%)	185 (0.48%)
>200	2 (0.63%)	120 (0.32%)
Aristolochic acid (mg) estimated total consumption		
0	270 (84.38%)	32550 (84.16%)
1–100	33 (10.31%)	5363 (13.09%)
101–200	4 (1.25%)	464 (1.20%)
201–300	4 (1.25%)	197 (0.51%)
>300	9 (2.81%)	.04%)

Analgesics *, sum of acetaminophen and non-steroidal anti-inflammatory drugs (NSAIDs).

Because only one UTC patient and 254 non-UTC controls had lived in area with endemic black foot disease (Table 1), we decided to exclude subjects with this characteristic in the final analysis for both cases and controls.

**Figure 1:**
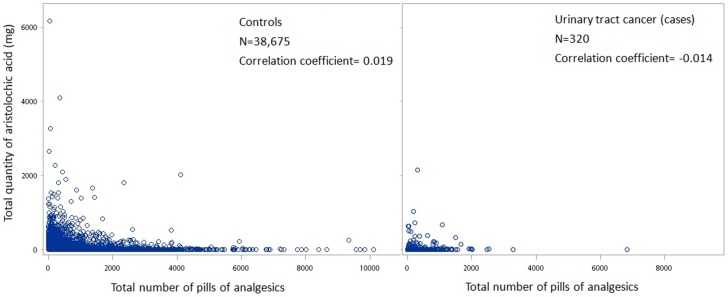
Correlation analysis between prescription of analgesics (number of pills) and cumulative dose of aristolochic acid for both cases and controls.

The number of new UTC cases and adjusted hazard ratios (aHR) calculated from the Cox regression models for multiple risk factors (sex, age, diabetes, hypertension, chronic UTI, analgesics and cumulative doses for different herbs) are summarized in Table 2. The crude and adjusted HR (aHR) for development of UTC increased significantly for older patients, whereas the crude and adjusted HR for development of UTC was not significantly related to either hypertension or diabetes. Crude HR's also increased for prescribed cumulative doses of Mu-Tong greater than 1 g, Fangchi 1–30 g, >100 g, as well as for prescribed Xi-Xin and for estimated AA consumptions of 1–100 mg, 101–200 g, and >300 mg. Prescription of analgesics also increased the crude HR. After control of potential confounding by other risk factors, we found that the aHR for development of UTC increased for ESRD patients prescribed Mu Tong, and that the various estimated consumptions of AA were each associated with an increased risk of UTC in the multivariable analyses (Mu Tong: at 1–30 g, aHR = 1.8, 95% CI = 1.3 to 2.6, and each 30 g increase, aHR = 1.3, 95% CI = 1.2 to 1.5; AA: at 1–100 mg, aHR = 2.1, 95% CI = 1.6 to 2.6, and each 100 mg increase, aHR = 1.6, 95% CI = 1.4 to 1.8). Prescription of analgesics is associated with a greater risk of urothelial cancer, with an increased aHR of 1.3 (95% CI = 1.2 to 1.4) for each increment of 150 pills, as summarized in models 1 and 2 of Table 2. However, there is little association between the prescribed numbers of analgesic pills and cumulative doses of AA, as shown in Figure 1.

**Table 2:** Crude and adjusted hazards ratios (HR), and 95% confidence intervals (CI) estimated from multivariate Cox regression models for urinary tract cancer developed in patients with ESRD.

		Model 1	Model 2
Risk Factors	Crude HR (95% CI)	Adjusted HR (95% CI)†	Adjusted HR (95% CI)†
Sex			
Men	1	1	1
Women	1.02 (0.82 to 1.27)	0.89 (0.71 to 1.12)	0.91 (0.73 to 1.14)
Age (years)			
<50	1	1	1
50–59	1.38 (1.04 to 1.85)*	1.43 (1.06 to 1.93)*	1.44 (1.07 to 1.92)*
60–69	1.77 (1.34 to 2.35)**	1.75 (1.30 to 2.36)**	1.73 (1.29 to 2.32)**
70–99	1.93 (1.39 to 2.66)**	1.90 (1.35 to 2.67)**	1.83 (1.31 to 2.56)**
Diabetes			
No	1	1	1
Yes	1.12 (0.90 to 1.40)	0.95 (0.76 to 1.20)	0.94 (0.75 to 1.19)
Hypertension			
No	1	1	1
Yes	1.00 (0.78 to 1.29)	0.74 (0.57 to 0.97)*	0.78 (0.60 to 1.01)
Chronic UTI			
No	1	1	1
Yes	4.77 (2.46 to 9.26)**	6.85 (3.52 to 13.34)**	6.68 (3.43 to 12.99)**
Analgesics (pills)			
0–150	1	1	1
151–300	2.03 (1.54 to 2.67)**	1.91 (1.44 to 2.53)**	1.93 (1.46 to 2.55)**
301–450	2.26 (1.55 to 3.28)**	1.97 (1.35 to 2.89)**	2.02 (1.39 to 2.95)**
451–600	2.92 (1.93 to 4.43)**	2.70 (1.75 to 4.15)**	2.72 (1.78 to 4.16)**
>600	3.36 (2.51 to 4.49)**	2.98 (2.19 to 4.06)**	2.83 (2.09 to 3.83)**
Each 150 pills increase	1.35 (1.26 to 1.44)**	1.32 (1.23 to 1.41)**	1.31 (1.22 to 1.40)**
Mu-Tong (g) total amount prescribed			
0	1	1	-
1–30	2.38 (1.79 to 3.16)**	1.83 (1.30 to 2.57)**	-
31–60	2.21 (1.29 to 3.82)‡	1.98 (1.07 to 3.69)*	-
61–100	3.08 (1.58 to 6.02)**	2.51 (1.19 to 5.28)*	-
101–200	2.73 (1.61 to 4.63)**	3.16 (1.63 to 6.14)**	-
>200	3.45 (1.88 to 6.36)**	3.46 (1.70 to 7.03)**	-
Each 30 g increase	1.33 (1.23 to 1.44)**	1.31 (1.17 to 1.48)**	-
Fangchi (g) total amount prescribed			
0	1	1	-
1–30	2.24 (1.73 to 2.92)**	1.23 (0.91 to 1.67)	-
31–100	1.41 (0.75 to 2.66)	0.84 (0.43 to 1.62)	-
>100	3.18 (1.31 to 7.70)*	1.89 (0.76 to 4.68)	-
Each 30 g increase	1.56 (1.33 to 1.84)**	1.22 (1.00 to 1.48)	
Xi-Xin (g) total amount prescribed			
0	1	1	-
1–30	2.02 (1.55 to 2.63)**	1.27 (0.91 to 1.78)	-
31–60	2.40 (1.44 to 3.99)**	1.50 (0.84 to 2.70)	-
61–100	2.90 (1.62 to 5.20)**	1.34 (0.69 to 2.60)	-
101–200	2.47 (1.26 to 4.83)‡	1.17 (0.53 to 2.59)	-
>200	2.25 (1.15 to 4.21)*	0.78 (0.34 to 1.81)	-
Each 30 g increase	1.28 (1.18 to 1.39)**	1.02 (0.89 to 1.16)	
Aristolochic acid (mg) estimated total consumption			
0	1	-	1
1–100	1.21 (0.83 to 1.78)	-	2.05 (1.61 to 2.60)**
101–200	2.16 (0.80 to 5.80)	-	2.84 (1.66 to 4.86)**
201–300	4.80 (1.79 to 12.91)‡	-	2.42 (0.89 to 6.55)
>300	6.29 (3.23 to 12.26)**	-	5.18 (2.86 to 9.40)**
Each 100 mg increase	1.56 (1.40 to 1.74)**	-	1.57 (1.40 to 1.75)**

† Logistic regression models for different dosages of Chinese herbs (model 1) and different estimated dosages of aristolochic acid as risk factors (model 2) were adjusted for age, sex, residence in township with endemic black foot disease, and history of chronic UTI.

*P<0.05.

‡ P<0.01.

**P<0.001.

## Discussion

To the best of our knowledge, this is the first population-based study to document a linear dose–response relationship between prescription of Chinese herbal products containing AA and the risk of UTC in ESRD patients after controlling for confounding by age, sex, living in a township endemic for black foot disease (a surrogate of arsenic contamination in the water supply), analgesic consumption, and history of chronic urinary tract infection. In fact, because the NHI reimbursement database collects all prescription information prospectively, we can rule out the possibility of recall bias for the intake doses of various Chinese herbal products. Since we included all ESRD patients newly diagnosed in Taiwan from 1998 to 2002, and the diagnosis of urinary tract cancer or end-stage renal disease made by doctors and officials of the NHI is usually accurate, we can also rule out the possibility of selection bias. Moreover, we excluded all subjects who had lived in townships endemic for black foot disease (a surrogate of high arsenic exposure) to prevent confounding the results due to the carcinogenic effects of arsenic exposure. Although increased prescription of analgesics was also associated with UTC (Table 2), the effect has been controlled in the multivariable regression model, and Figure 1 also shows no association between the number of analgesics pills and cumulative dose of AA. Finally, this study has documented a dose-dependent association between the cumulative estimated prescribed dose of AA and urinary tract cancer, as well as a dose-dependent association between the cumulative prescribed dose of Mu-Tong and UTC. We thus tentatively conclude that the urothelial cancers developed by ESRD patients are associated with prescription of AA-associated Chinese herbal products.

This study found a consistent dose-response relationship between the estimated intake of AA (or prescribed dose of AA-containing CHP) and urinary tract cancer in ESRD patients, suggesting that AA may be responsible for increased cancer risk of these patients. Fangchi and Xi-Xin both showed increased hazard ratios at higher doses, but these results did not reach statistical significance after adjustment for risk factors, likely due to the small number of case subjects. However, the increased hazard ratios for the occurrence of UC were found to be significantly higher in ESRD patients when over 1–30 g of Mu-Tong or over 1–100 mg cumulative AA were prescribed. Because these doses are much smaller than those reported by Belgian scholars [4] and our previous report [7], it suggests that patients with ESRD might be more vulnerable to the carcinogenic effects of AA. Although these subgroups do not correlate with a cumulative dose of higher than 147-mg AA, as reported in the Belgian report [4], the observation of increased hazard ratios at lower cumulative levels of AA-containing Chinese herbal products in ESRD patients who consume these products suggests potential pathogenic effects at lower doses, and this is an issue that deserves further investigation, with more long-term follow-up of these patients.

Forty-five percent of the UTC cases in this study were upper urinary tract cancer, which is similar to the rates seen in the general population, as reported by the National Cancer Registry and in a previous clinical report examining pathology-confirmed urinary tract cancer cases in Taiwan [41]. However, these rates are much higher than those in other countries, in which less than 10% of all UTC cases are upper urinary tract cancer. In this study, prescription of Chinese herbal products was associated with urothelial cancers that occurred in all parts of the urinary tract, similar to what was reported in a recent case study of Belgian women who received kidney transplants for end-stage AA nephropathy, in which 44.7% had upper urinary tract cancer and 39.5% had bladder cancer [42]. We thus hypothesize that AA induces urothelial cancers in the upper urinary tract and bladder with approximately equal tendency.

We also found a dose-dependent association between analgesics and occurrence of UTC among patients with ESRD, corroborating previous reports [17]–[19]. Unfortunately, we did not have a sufficiently large sample size to further explore this issue. Future studies are thus recommended to collect more UTC cases among patients with ESRD, and determine if the effect is associated with acetaminophen, aspirin, or any other NSAID.

There are some limitations to this study, as follows. First, because patient identities were not obtainable from the NHI reimbursement database, histopathology reports were unavailable to confirm the diagnoses. However, accurate diagnosis of UTC in the NHI database is based on pathology and/or cytology evidence and made after serious consideration with histopathologic proof in 95% of bladder cancers and 91%–92% of upper urinary tract cancers [43]. Second, we were unable to contact patients directly about their use of herbs due to anonymization of the database; therefore, we were unable to rule out the possibility that subjects may have taken additional nephrotoxic herbs or agents that were not prescribed. However, the comprehensive coverage and copayment for prescriptions is universally 50 NT$ (approximately equal to US $1.5), which is generally less than the cost of herbs sold in Taiwan's markets. It is thus unlikely that the subjects purchased AA-containing herbs or nephrotoxic drugs without a prescription. Third, we also could not validate the actual intake of prescribed herbal product by the patients. Because 95% of the dosing frequenciesfor Chinese herbal products last for only one week [44], a large cumulative dose indicates that patients on long-term prescriptions actually consumed the prescribed medication. However, if the patients did not take all of the prescribed medication, our findings would underestimate the effects of AA-related Chinese herbal consumption. Fourth, because the NHI data did not include smoking history, we could not control for this variable in our models. However, because smoking rates in Taiwan in the last two decades have ranged from 47% to 62% and from 2.3% to 5.3%, for males and females, respectively, male patients with ESRD would be expected to have a higher risk of developing UTC if smoking were a major contributing risk factor [45]. As we did not find any increased risk of UTC in males compared to females, our results do not seem to be confounded by smoking.

## Conclusions

This study finds that AA from Chinese herbal products and analgesics is associated with increased risk of developing UTC in ESRD patients, due to these having been prescribed low doses of Mu-Tong. The linear dose-response relationships found in this work may be useful in consideration of a total ban or establishment of limits on the consumption of such herbal products among patients with ESRD and/or chronic kidney disease. More studies are needed to examine the potential carcinogenic effects of analgesics on patients with ESRD and/or chronic renal failure. In addition, regular follow-up screening for UC in ESRD patients who have consumed AA-related Chinese herbal products is also necessary.

## Supporting Information

File S1 **The commercial names of 45 kinds of non-steroidal anti-inflammatory drugs in the reimbursement database.**
(DOCX)

## References

[1] Vanherweghem JL, Depierreux M, Tielemans C, Abramowicz D, Dratwa M, et al (1993) Rapidly progressive interstitial renal fibrosis in young women: association with slimming regimen including Chinese herbs. Lancet 341: 387–391. 8094166 10.1016/0140-6736(93)92984-2

[2] Vanhaelen M, Vanhaelen-Fastre R, But P, Vanherweghem JL (1994) Identification of aristolochic acid in Chinese herbs. Lancet 343: 174. 10.1016/s0140-6736(94)90964-4 7904018

[3] Cosyns JP (2003) Aristolochic acid and ‘Chinese herbs nephropathy’: a review of the evidence to date. Drug safety : an international journal of medical toxicology and drug experience 26: 33–48. 10.2165/00002018-200326010-00004 12495362

[4] Nortier JL, Martinez MC, Schmeiser HH, Arlt VM, Bieler CA, et al (2000) Urothelial carcinoma associated with the use of a Chinese herb (Aristolochia fangchi). N Engl J Med 342: 1686–1692. 10841870 10.1056/NEJM200006083422301

[5] Cosyns JP, Jadoul M, Squifflet JP, Wese FX, van Ypersele de Strihou C (1999) Urothelial lesions in Chinese-herb nephropathy. American journal of kidney diseases : the official journal of the National Kidney Foundation 33: 1011–1017. 10352187 10.1016/S0272-6386(99)70136-8

[6] Arlt VM, Stiborova M, Schmeiser HH (2002) Aristolochic acid as a probable human cancer hazard in herbal remedies: a review. Mutagenesis 17: 265–277. 12110620 10.1093/mutage/17.4.265

[7] Lai MN, Wang SM, Chen PC, Chen YY, Wang JD (2010) Population-based case-control study of Chinese herbal products containing aristolochic acid and urinary tract cancer risk. J Natl Cancer Inst 102: 179–186. 20026811 10.1093/jnci/djp467 PMC2815723

[8] Lai MN, Wang SM, Chen PC, Chen YY, Wang JD (2009) Population-based case-control study of Chinese herbal products containing aristolochic acid and urinary tract cancer risk. J Natl Cancer Inst 102: 179–186. 20026811 10.1093/jnci/djp467 PMC2815723

[9] Matas AJ, Simmons RL, Kjellstrand CM, Buselmeier TJ, Najarian JS (1975) Increased incidence of malignancy during chronic renal failure. Lancet 1: 883–886. 47534 10.1016/s0140-6736(75)91684-0

[10] Port FK, Ragheb NE, Schwartz AG, Hawthorne VM (1989) Neoplasms in dialysis patients: a population-based study. American journal of kidney diseases : the official journal of the National Kidney Foundation 14: 119–123. 2787957 10.1016/s0272-6386(89)80187-8

[11] Kjellstrand CM (1979) Are malignancies increased in uremia? Nephron 23: 159–161. 471138 10.1159/000181627

[12] Maisonneuve P, Agodoa L, Gellert R, Stewart JH, Buccianti G, et al (1999) Cancer in patients on dialysis for end-stage renal disease: an international collaborative study. Lancet 354: 93–99. 10408483 10.1016/s0140-6736(99)06154-1

[13] Chuang CH, Lee CT, Tsai TL, Chen JB, Hsu KT, et al (2002) Urological malignancy in chronic dialysis patients. Acta Nephrologica 16: 19–24.

[14] Chang CH, Yang CM, Yang AH (2007) Renal diagnosis of chronic hemodialysis patients with urinary tract transitional cell carcinoma in Taiwan. Cancer 109: 1487–1492. 17330839 10.1002/cncr.22557

[15] Ou JH, Pan CC, Lin JS, Tzai TS, Yang WH, et al (2000) Transitional cell carcinoma in dialysis patients. European urology 37: 90–94. 10671792 10.1159/000020106

[16] Chen KS, Lai MK, Huang CC, Chu SH, Leu ML (1995) Urologic cancers in uremic patients. American journal of kidney diseases : the official journal of the National Kidney Foundation 25: 694–700. 7747722 10.1016/0272-6386(95)90544-8

[17] Gonwa TA, Corbett WT, Schey HM, Buckalew VM Jr (1980) Analgesic-associated nephropathy and transitional cell carcinoma of the urinary tract. Annals of internal medicine 93: 249–252. 7406375 10.7326/0003-4819-93-2-249

[18] Swindle P, Falk M, Rigby R, Petrie J, Hawley C, et al (1998) Transitional cell carcinoma in renal transplant recipients: the influence of compound analgesics. British journal of urology 81: 229–233. 9488064 10.1046/j.1464-410x.1998.00496.x

[19] Kliem V, Thon W, Krautzig S, Kolditz M, Behrend M, et al (1996) High mortality from urothelial carcinoma despite regular tumor screening in patients with analgesic nephropathy after renal transplantation. Transplant international : official journal of the European Society for Organ Transplantation 9: 231–235. 8723192 10.1007/BF00335391

[20] Vanherweghem LJ (1998) Misuse of herbal remedies: the case of an outbreak of terminal renal failure in Belgium (Chinese herbs nephropathy). Journal of alternative and complementary medicine 4: 9–13. 9553830 10.1089/acm.1998.4.1-9

[21] Chiang HS, Guo HR, Hong CL, Lin SM, Lee EF (1993) The incidence of bladder cancer in the black foot disease endemic area in Taiwan. British journal of urology 71: 274–278. 8477313 10.1111/j.1464-410x.1993.tb15942.x

[22] Chiou HY, Chiou ST, Hsu YH, Chou YL, Tseng CH, et al (2001) Incidence of transitional cell carcinoma and arsenic in drinking water: a follow-up study of 8,102 residents in an arseniasis-endemic area in northeastern Taiwan. American journal of epidemiology 153: 411–418. 11226969 10.1093/aje/153.5.411

[23] Wiessler M (1994) DNA adducts of pyrrolizidine alkaloids, nitroimidazoles and aristolochic acid. IARC Sci Publ 165–177. 7806311

[24] TaiwanYearbook2009 Public Health: Health Insurance.

[25] NHRI-Taiwan (2003) National Health Research Database.

[26] Centers_for_Disease_Control_and_Prevention (2009) International Classification of Diseases, Ninth Revision (ICD-9). Atlanta, Georgia.

[27] Perneger TV, Whelton PK, Klag MJ (1994) Risk of kidney failure associated with the use of acetaminophen, aspirin, and nonsteroidal antiinflammatory drugs. N Engl J Med 331: 1675–1679. 7969358 10.1056/NEJM199412223312502

[28] Gago-Dominguez M, Yuan JM, Castelao JE, Ross RK, Yu MC (1999) Regular use of analgesics is a risk factor for renal cell carcinoma. Br J Cancer 81: 542–548. 10507783 10.1038/sj.bjc.6690728 PMC2362920

[29] Committee Chinese Medicine and Pharmacy DOH-T (2002) Unified Formulas.

[30] Hsu Y, Tseng H, Wen K (1997) Determination of aristolochic acid in Fangchi radix. Ann Rept NLFD Taiwan ROC 136–142 (In Chinese).

[31] Tung C, Ho Y, Tsai H, Chong Y (1999) Studies on the commonly musused and adulterated Chinese crude drug species in Taiwan. Chin Med Coll J 35–46.

[32] Deng J (2002) Quality evaluation of Fang-Ji and analysis of marker constituents [dissertation]. Taiwan: Institute of Chiense Pharmaceutical Sciences, China Medical University 75–77.

[33] Chuang M, Hsu Y, Chang H, Lin J, Liao C (2002) Studies on adulteration and misusage of marketed Akebiae caulisn. Ann Rept NLFD Taiwan ROC 104–119 (In Chinese).

[34] Jong TT, Lee MR, Hsiao SS, Hsai JL, Wu TS, et al (2003) Analysis of aristolochic acid in nine sources of Xixin, a traditional Chinese medicine, by liquid chromatography/atmospheric pressure chemical ionization/tandem mass spectrometry. J Pharm Biomed Anal 33: 831–837. 14623612 10.1016/s0731-7085(03)00310-8

[35] Hsu Y, Lo C, Chang H, Lin J (2003) Studies on adulteration and misusage of Asari radi in the market. Ann Rept NLFD Taiwan ROC 153–167 (In Chinese).

[36] Chen CJ, Chuang YC, Lin TM, Wu HY (1985) Malignant neoplasms among residents of a blackfoot disease-endemic area in Taiwan: high-arsenic artesian well water and cancers. Cancer research 45: 5895–5899. 4053060

[37] Chen CJ, Chuang YC, You SL, Lin TM, Wu HY (1986) A retrospective study on malignant neoplasms of bladder, lung and liver in blackfoot disease endemic area in Taiwan. British journal of cancer 53: 399–405. 3964542 10.1038/bjc.1986.65 PMC2001352

[38] Johansson SL, Cohen SM (1997) Epidemiology and etiology of bladder cancer. Seminars in surgical oncology 13: 291–298. 9259084 10.1002/(sici)1098-2388(199709/10)13:5<291::aid-ssu2>3.0.co;2-8

[39] Groah SL, Weitzenkamp DA, Lammertse DP, Whiteneck GG, Lezotte DC, et al (2002) Excess risk of bladder cancer in spinal cord injury: Evidence for an association between indwelling catheter use and bladder cancer. Archives of Physical Medicine and Rehabilitation 83: 346–351. 11887115 10.1053/apmr.2002.29653

[40] Jiang X, Castelao JE, Groshen S, Cortessis VK, Shibata D, et al (2009) Urinary tract infections and reduced risk of bladder cancer in Los Angeles. British journal of cancer 100: 834–839. 19174821 10.1038/sj.bjc.6604889 PMC2653778

[41] (2008) Cancer Incidence Rate in Taiwan, 1998–2002 & 2003–2007 http://tcr.cph.ntu.edu.tw/main.php?Page=N2: Taiwan cancer registry.

[42] Achenbach H, Fischer A (1997) 6-O-beta-D-glucoside of aristolochic acid IIIa and other components from the roots of Aristolochia baetica. Planta Med 63: 579. 17252385 10.1055/s-2006-957777

[43] TaiwanCancerRegistry (2009) Cancer incidence rate in Taiwan, 1998–2002.

[44] Hsieh SC, Lai JN, Lee CF, Hu FC, Tseng WL, et al (2008) The prescribing of Chinese herbal products in Taiwan: a cross-sectional analysis of the national health insurance reimbursement database. Pharmacoepidemiology and drug safety 17: 609–619. 18481335 10.1002/pds.1611

[45] (2013) Adult Smoking Behavior Surveillance System,ASBS. Taipei, Taiwan.

