# Supplementary material for: Increased Risk of Urinary Tract Cancer in ESRD Patients Associated with Usage of Chinese Herbal Products Suspected of Containing Aristolochic Acid
Source: PLoS One. 2014 Aug 29;9(8):e105218. doi: 10.1371/journal.pone.0105218 (PMC4149424; doi:10.1371/journal.pone.0105218)
Supplement: File S1 — The commercial names of 45 kinds of non-steroidal anti-inflammatory drugs in the reimbursement database. (DOCX) [file pone.0105218.s001.docx]

The commercial names of 45 kinds of non-steroidal anti-inflammatory drugs in the reimbursement database: acemetacin; alclofenac; alminoprofen; apronalide; avapyrazone; benzydamine; celecoxib; diclofenac; diflunisal; dipyrone (sulpyrin); ephedryl isopropylantipyrine; ethenzamide; etodolac; etofenamate; fenbufen; fenoprofen; flufenamate; flurbiprofen; glafenine; hydroxybutyric acid b-p-phenetidide; ibuprofen; indomethacin; ketoprofen; ketorolac tromethamine; meclofenamate; mefenamic acid; meloxicam; mepirizole; nabumetone; naproxen; niflumic acid; nimesulide; pantopon; phenylbutazone; piroxicam; propyphenazone; proxazole citrate; pyrabital; salsalate; sulindac; tenoxicam; tiaprofenic acid; tiaramide; tolfenamic acid; tolmetin.
